# Evaluation of Mother-Child Agreement and Factorial Structures of the SCARED Questionnaire in an Italian Clinical Sample

**DOI:** 10.3389/fpsyg.2017.00242

**Published:** 2017-02-24

**Authors:** Simona Scaini, Anna Ogliari, Ludovica De Carolis, Laura Bellodi, Clelia Di Serio, Chiara Brombin

**Affiliations:** ^1^Faculty of Psychology, Sigmund Freud UniversityMilan, Italy; ^2^Developmental Psychopathology Unit, Vita-Salute San Raffaele UniversityMilan, Italy; ^3^Department of Clinical Neuroscience, San Raffaele Scientific InstituteMilan, Italy; ^4^Faculty of Psychology, Vita-Salute San Raffaele UniversityMilan, Italy; ^5^University Centre of Statistics in the Biomedical Sciences, Vita-Salute San Raffaele UniversityMilan, Italy

**Keywords:** anxiety, assessment, factor analysis, internalizing disorders, rating scales

## Abstract

**Background:** A great part of the literature has confirmed the importance of both child and parents reports as source of factual information, especially for childhood emotional syndromes. In our study we aimed at: (i) calculating mother-child agreement and (ii) evaluating factorial structure of the Screen for Child Anxiety Related Emotional Disorders (SCARED) questionnaire in an Italian clinical sample. The novelty of this contribution is two-fold: first, from a clinical point of view, we investigated the parent-child agreement level and examined separately the factorial structures of both parent and child versions of the SCARED for the first time in an Italian clinical sample. Second, unlike previous studies, we used statistical approaches specifically suited to account for the ordinal nature of the collected variables.

**Method:** In a clinical sample of 171 children and adolescents aged 8–18 and their mothers we evaluated inter-rater agreement using weighted kappa indices to assess agreement for each item belonging to a certain SCARED subscale. Exploratory factor analysis for ordinal data was then performed on the polychoric correlation matrix calculated on SCARED items. Differences in the numbers of symptoms reported by children and parents were evaluated as well.

**Results and Conclusions:** Our results reveal moderate to strong mother-child agreement. A significant age effect is present. Two different factorial solutions emerged for parent and child SCARED versions (a 5 factor structure for parents and a 6 factor solution in the child version, including a new factor “Worry about Parents”). This study confirmed the importance of evaluating both child and parent reports in assessment protocols for anxiety disorders. Our findings could help clinicians to determine which information, and from which rater, must be accounted for in evaluating treatment decisions. Moreover, we find that patients characteristics, such as gender and age, should be taken into account when assessing agreement.

## Introduction

Anxiety disorders are the most common psychiatric illnesses in both adulthood and childhood (Beesdo-Baum and Knappe, 2012; Kessler et al., 2012). Anxiety symptoms tend to begin early in life and are likely to become chronic and persistent (Beesdo et al., 2009). Thus, early recognition and treatment are desirable (Beidel and Turner, 2007; Davis et al., 2011; Scaini et al., 2016). However, since anxious children do not manifest behavioral difficulties as clearly as children with externalizing disorders, they often go unrecognized and underdiagnosed (Costello et al., 2005; Beesdo et al., 2009).

In light of these considerations, it is important to find new adequate and validated instruments to screen the severity of anxiety symptoms in childhood. In addition to structured psychiatric interviews, which are specific and detailed, questionnaires may be used as flexible, easy to administer, less costly, and less time consuming first screening instruments (Verhulst and Van der Ende, 2006; Garcia-Lopez et al., 2015). Several anxiety self-report questionnaires have been developed to assess anxiety symptoms in both clinical and general population. In a review on anxiety rating scales, Myers and Winters (2002) reported that the Screen for Child Anxiety Related Emotional Disorders (SCARED) is one of the best self-report instrument to evaluate anxiety symptoms. Moreover, a meta-analysis by Hale et al. (2011) suggested that the SCARED presents good psychometric properties and can be used as a cross-cultural screening instrument. The SCARED offers both self- and a parent-report version to assess childhood anxiety disorders by including subscales on General Anxiety, Separation Anxiety, Somatic/Panic, Social Anxiety, and School Phobia. Several reports have already investigated psychometric properties in community and clinical populations reporting good internal consistency, test-retest reliability, and discriminant validity (Birmaher et al., 1997, 1999; Ogliari et al., 2006; Crocetti et al., 2009; Hale et al., 2013).

A great part of the literature has confirmed the importance of both child and parent report as sources of factual information, especially for childhood emotional syndromes. Indeed, when using rating scales, it is crucial that clinicians collect information from different sources. The information provided by several raters, which see children in different contexts, is important to obtain a comprehensive picture of the child's functioning (Verhulst and Van der Ende, 2006). As reported by Verhulst and Van der Ende (2006) parents are usually familiar with their child's functioning in many situations and throughout time. However, children and adolescents' self-reports are also indispensable, especially to have a better overview of internalizing problems that are more difficult to detect (Costello et al., 2005; Beesdo et al., 2009).

Previous studies using different methods and measures indicated low levels of agreement among informants, showing higher scores for anxiety, and depressive symptoms in child report than in parent report (De Los Reyes and Kazdin, 2005; Salbach-Andrae et al., 2009).

Besides focusing on low agreement levels as a nuisance source (Verhulst and Van der Ende, 2006), in existing works it has been reported the importance of multiple informants as a potentially valid source of information (Kraemer et al., 2003). Self-reports filled in by parents and children are now regarded as being of equal weight in the diagnostic process (Angold, 2002). Several reports showed that parents perception of anxiety may be influenced by cultural factors (Gaines et al., 1997; Murphy et al., 2003; Wren et al., 2007).

Until now, the agreement of the Italian child and parent versions of SCARED has not been examined. This lack calls for a systematic analysis of parent-child agreement in the Italian culture. Moreover, the possible effect of sex and age on agreement was investigated. Better knowledge of the impact of these factors could help in shaping more effective assessment protocols and treatments. For instance, information on sex and age could encourage clinicians in considering also these variables in their clinical decisions (Rutter et al., 2008).

To the best of our knowledge, no previous studies have analyzed parent-child agreement using indices suited for ordinal variables such as κ index. Moreover, the level of agreement (taking into account age and sex effect) for each item within each SCARED subscale has not been addressed in the previous literature so far.

Indeed, all recent studies that have evaluated the psychometric properties of the SCARED questionnaire (e.g., Wren et al., 2007; Su et al., 2008; Jastrowski Mano et al., 2012) in exploratory fashion, applied a principal component analysis without specifying on which correlation matrix (Pearson, Spearman, or polychoric correlation matrix) was the analysis carried out. In the common practice, ordinal variables with at least five categories are treated as continuous variables and analyzed with a traditional factor analysis procedure, thus ignoring the ordinal nature of the outcomes. Treating nominal and ordinal data as interval or ratio data, may lead to unreliable parameter estimates as well as to poor goodness of fit, especially in presence of highly skewed non Gaussian data or polarized/bimodal distributions (Muthén and Kaplan, 1985).

As emphasized in Holgado-Tello et al. (2010) in presence of ordinal data, factor analysis should be carried out on the matrix of polychoric correlations rather than on Pearson correlation matrix since analyses based on the former produce a more accurate reproduction of the original correlation structure.

Motivated by the outlined importance of accounting for different sources of information in screening for anxiety disorders, we investigate the parent-child agreement level and examined separately the factorial structures of both parent and child versions of the SCARED. For the first time in the literature we have carried out the analysis in an Italian clinical sample.

In particular, we analyze the cross-informant agreement levels for each questionnaire item and, considering age and sex effect, for each factor-based scales. We, then inspect the differences in the number of symptoms reported by children and parents. Lastly, we examine separately the factor structures of parent- and self-report versions of SCARED questionnaire.

## Materials and methods

### Participants and procedure

Two hundred and ten children and adolescents (54% boys and 46% girls) who were referred to an outpatient treatment center of Development and Psychopathology Unit at San Raffaele Hospital filled in the SCARED. Mean age of the sample was 12.0 years (*SD* 2.9, range 7–17 years). Most children (>90%) were Caucasian. Children and mothers completed the SCARED in the lab after having received their first neuropsychiatric visit. During the questionnaire compilation, a clinical psychologist remains available to answer questions. Then, the Kiddie-Sads-Present and Lifetime Version (K-SADS-PL, Kaufman et al., 1997) was administered by clinical psychologists to diagnose psychiatric disorder. None of the children involved in this study met the full criteria for a current or past diagnosis of psychiatric or neurological disorder. However, clinical assessment revealed the presence of subclinical psychiatric symptoms in the sample (27% of the children manifested symptoms of Affective Disorders, 15% of Anxiety Disorders, 16% of Attention Deficit/Hyperactivity Disorder, 14% of Oppositional Defiant Disorder, and 16% of Conduct Disorder).

The study was conducted according to the Declaration of Helsinki. Data were collected during clinical assessment and parents gave their informed consent to use, anonymously, data for research purposes. According to the hospital's ethics committee guidelines, this type of study did not require an approval by the institutional review board. The head physician of the Development and Psychopathology Unit at San Raffaele Hospital and the dean of the Faculty of Psychology at San Raffaele University examined and approved the study.

### Measures

#### Screen for child anxiety related emotional disorders (SCARED)

Children and their mothers filled in the Italian version of the 41-item SCARED questionnaire (Ogliari et al., 2006). The SCARED questionnaire was originally devised to screen Anxiety Disorders (AD) in clinical samples (Birmaher et al., 1997, 1999; Ogliari et al., 2006; Crocetti et al., 2009), but it has been also employed as valuable screening tool in community samples (e.g., Boyd et al., 2003; Crocetti et al., 2009). Children were asked to rate the frequency with which they experienced each symptom on a 3-point likert scale (0 = “almost never,” 1 = “sometimes,” 2 = “often”). The questionnaire was completed also by their mothers who were asked to rate the frequency with which their children experienced each symptom on the same scale. Via principal component factor analysis, Birmaher et al. (1997), identified five subscales, i.e., Panic/Somatic Anxiety (PD), General Anxiety (GAD), Separation Anxiety (SAD), Social Phobia (SOC), and School Phobia (SCH). With reference to the psychometric properties, the tool showed good internal consistency, test-rest reliability, discriminative validity. The authors reported moderate parent-child agreement (ρ = 0.20–0.47).

When a cut-off point of 25 was applied to the total score endorsed by subjects across these five factors, data showed good sensitivity (70%) and good ability to discriminate between children with AD versus those without AD (specificity: 67%), and between children with AD versus those with depression, or disruptive disorders: 61 and 71%, respectively; (Birmaher et al., 1999; Monga et al., 2000). Finally, in a meta-analytic study, Hale et al. (2011) found that internal consistency for the total score was good (α = 0.89–0.91) although substantial variation was found in the internal consistency of the subscales (α = 0.43–0.93).

### Statistical analyses

We used a weighted kappa index with “squared” weights (Cohen, 1960; Fleiss et al., 2013) to evaluate chance-corrected mother-child agreement for each item belonging to a certain subscale. The weighted version of this index has been introduced by Cohen (1968) as a method to deal with ordinal data.

While traditional kappa does not distinguish between disagreements, treating them on an equal footing (Banerjee et al., 1999), there are circumstances (e.g., when making diagnosis) where some disagreements have more severe implications than others. Hence, a weighted version should be preferred in such cases. By choosing the “squared” weight option, disagreements are weighted according to their squared distance from perfect agreement.

Spearman correlation coefficients were calculated to examine mother-child agreement on the five SCARED subscales.

Exploratory factor analysis for ordinal data was performed on polychoric correlation matrices calculated on the SCARED items (Gilley and Uhlig, 1993; Jöreskog and Moustaki, 2001). Ordinary Least Squares (OLS) procedure that minimizes the residual (off-diagonal) correlation matrix (minres algorithm; Harman and Jones, 1966) was applied to extract factors.

Parent and child versions were analyzed separately to uncover potential differences among emerging factorial structures.

In order to select the number of factors to retain in the factorial model, several statistical criteria have been considered. In particular, we focused on Horn's Parallel Analysis (Horn, 1965), Velicer's Minimum Average Partial criterion (MAP; Velicer, 1976), the Very Simple Structure criterion (VSS; Revelle and Rocklin, 1979) as well as the empirically derived Bayesian Information Criterion (eBIC).

Goodness of model fit has been examined through the Root Mean Square of the Residuals (RMSR) that evaluates the difference between the observed correlation matrix and the estimated one as reproduced by the factorial model. The choice of the proposed factorial solution was led by considerations on the total amount of variance explained, the number of variables that load on each factor and parsimony of the representation.

Analyses were performed using R Statistical Software (R Development Core Team, 2014), version 3.3.1. In particular, *psych* (Revelle, 2014) and *irr* (Gamer et al., 2012) packages were used to implement factor analyses on polychoric correlation matrices and to calculate coefficients of inter-rater agreement, respectively.

## Results

### Evaluation of inter-rater agreement

Mother-child agreement on SCARED subscales was assessed by computing Spearman bivariate correlations between subscale scores (Table 1). Correlation strength has been interpreted according to Bartz (1999).

**Table 1:** **Mean scores for each SCARED subscale and for each perspective along with mother-child agreement assessed through Spearman correlations**.

	**Children**	**Mother**	**Cross-informant agreement**
**Overall**	***n** = **171***	***n** = **150***	**Child-Mother (*****n** = **133*****)**
Somatic/panic anxiety	5.73 (4.91)	4.35 (4.57)	0.56
General anxiety	7.68 (4.56)	7.43 (4.36)	0.48
Separation anxiety	5.04 (3.47)	5.17 (3.88)	0.67
Social phobia	5.87 (3.68)	5.19 (3.96)	0.66
School phobia	2 (1.95)	2.03 (2.03)	0.63
Total	26.32 (13.56)	24.17 (14.2)	0.6
**Group 8–10**	***n*** = **49**	***n*** = **49**	**Child-Mother (*****n** = **40*****)**
Somatic/panic anxiety	4.84 (5.09)	3.59 (4.15)	0.48
General anxiety	5.9 (3.86)	7.18 (4.19)	0.49
Separation anxiety	7.24 (3.56)	6.61 (3.63)	0.61
Social phobia	5.29 (3.41)	3.92 (3.21)	0.41
School phobia	1.61 (1.77)	1.53 (1.7)	0.71
Total	24.88 (12.98)	22.84 (12.11)	0.36
**Group 11–13**	***n** = **53***	***n** = **45***	**Child-Mother (*****n** = **40*****)**
Somatic/panic anxiety	5.11 (3.79)	4.4 (4.58)	0.55
General anxiety	7.11 (4.16)	7.89 (4.43)	0.41
Separation anxiety	4.49 (2.93)	5.02 (3.92)	0.62
Social phobia	5.58 (3.62)	5.47 (4.14)	0.79
School phobia	1.94 (2)	2.04 (2.23)	0.55
Total	24.25 (12.07)	24.82 (13.98)	0.64
**Group 14–15**	***n** = **34***	***n** = **30***	**Child-Mother (*****n** = **28*****)**
Somatic/panic anxiety	4.85 (3.81)	3.53 (4.04)	0.52
General anxiety	8.26 (4.72)	7.07 (4.31)	0.61
Separation anxiety	3.47 (2.72)	3.63 (3.41)	0.51
Social phobia	6.91 (3.9)	5.9 (3.98)	0.71
School phobia	2.24 (1.99)	2.1 (2.01)	0.58
Total	25.74 (13.63)	22.23 (14.87)	0.69
**Group 16–18**	***n** = **35***	***n** = **26***	**Child-Mother (*****n** = **25*****)**
Somatic/panic anxiety	8.77 (5.98)	6.65 (5.33)	0.6
General anxiety	10.46 (4.62)	7.54 (4.77)	0.57
Separation anxiety	4.31 (3.38)	4.5 (4.06)	0.75
Social phobia	6.11 (3.83)	6.27 (4.48)	0.69
School phobia	2.4 (2.03)	2.85 (2.13)	0.67
Total	32.06 (15.31)	27.81 (17.27)	0.72
**Female**	***n** = **82***	***n** = **70***	**Child-Mother (*****n** = **64*****)**
Somatic/panic anxiety	6.6 (5.14)	4.23 (4.41)	0.57
General anxiety	8.66 (4.76)	7.27 (4.21)	0.47
Separation anxiety	5.13 (3.17)	4.94 (3.92)	0.67
Social phobia	6.04 (3.63)	5.29 (3.99)	0.66
School phobia	2.27 (1.98)	2.06 (2.06)	0.65
Total	28.7 (12.87)	23.79 (13.31)	0.53
**Male**	***n** = **89***	***n** = **80***	**Child-Mother (*****n** = **69*****)**
Somatic/panic anxiety	4.93 (4.57)	4.46 (4.74)	0.53
General anxiety	6.78 (4.19)	7.58 (4.5)	0.53
Separation anxiety	4.96 (3.74)	5.38 (3.87)	0.68
Social phobia	5.72 (3.74)	5.1 (3.96)	0.66
School phobia	1.75 (1.89)	2 (2.02)	0.62
Total	24.13 (13.88)	24.51 (15.02)	0.65

Overall (i.e., considering all subjects regardless of children age, after pairwise deletion, in a sample of *n* = 133) all correlations were from moderate to strong (*r* ranges from 0.48 to 0.67, PD scale *r* = 0.56, GAD scale *r* = 0.48, SAD scale *r* = 0.67, SOC scale *r* = 0.66, SCH scale *r* = 0.63, all statistically significant).

In order to assess agreement invariance across ages, four age groups have been defined (8–10 yrs, *n*= 66; 11–13 yrs, *n* = 62; 14–15 yrs, *n* = 40; 16–18 yrs, *n* = 41, 1 missing data) representing different school grades and stages of development. All correlations related to the first group (8–10 yrs, *n* = 40 after pairwise deletion), ranged from moderate to strong (*r* ranges from 0.41 to 0.71, PD scale *r* = 0.48, GAD scale *r* = 0.49, SAD scale *r* = 0.61, SOC scale *r* = 0.41, SCH scale *r* = 0.71, all significant).

In the second group (11–13 yrs, *n* = 40 after pairwise deletion), the mother-child agreement *r* ranges from 0.41 to 0.79 (PD scale *r* = 0.55, GAD scale *r* = 0.41, SAD scale *r* = 0.62, SOC scale *r* = 0.79, SCH scale *r* = 0.55, all significant).

The third group of students, aged between 14 and 15 years (*n* = 28 after pairwise deletion), shows correlations varying from moderate to strong range (*r* ranges from 0.51 to 0.71, PD scale *r* = 0.52, GAD scale *r* = 0.61, SAD scale *r* = 0.51, SOC scale *r* = 0.71, SCH scale *r* = 0.58, all significant).

Similar correlations were found in the last group, including adolescents aged between 16 and 18 years (*n* = 25 after pairwise deletion) with *r* ranging from 0.57 to 0.75 (PD scale *r* = 0.60, GAD scale *r* = 0.57, SAD scale *r* = 0.75, SOC scale *r* = 0.69, SCH scale *r* = 0.67, all significant).

**Figure 1:**
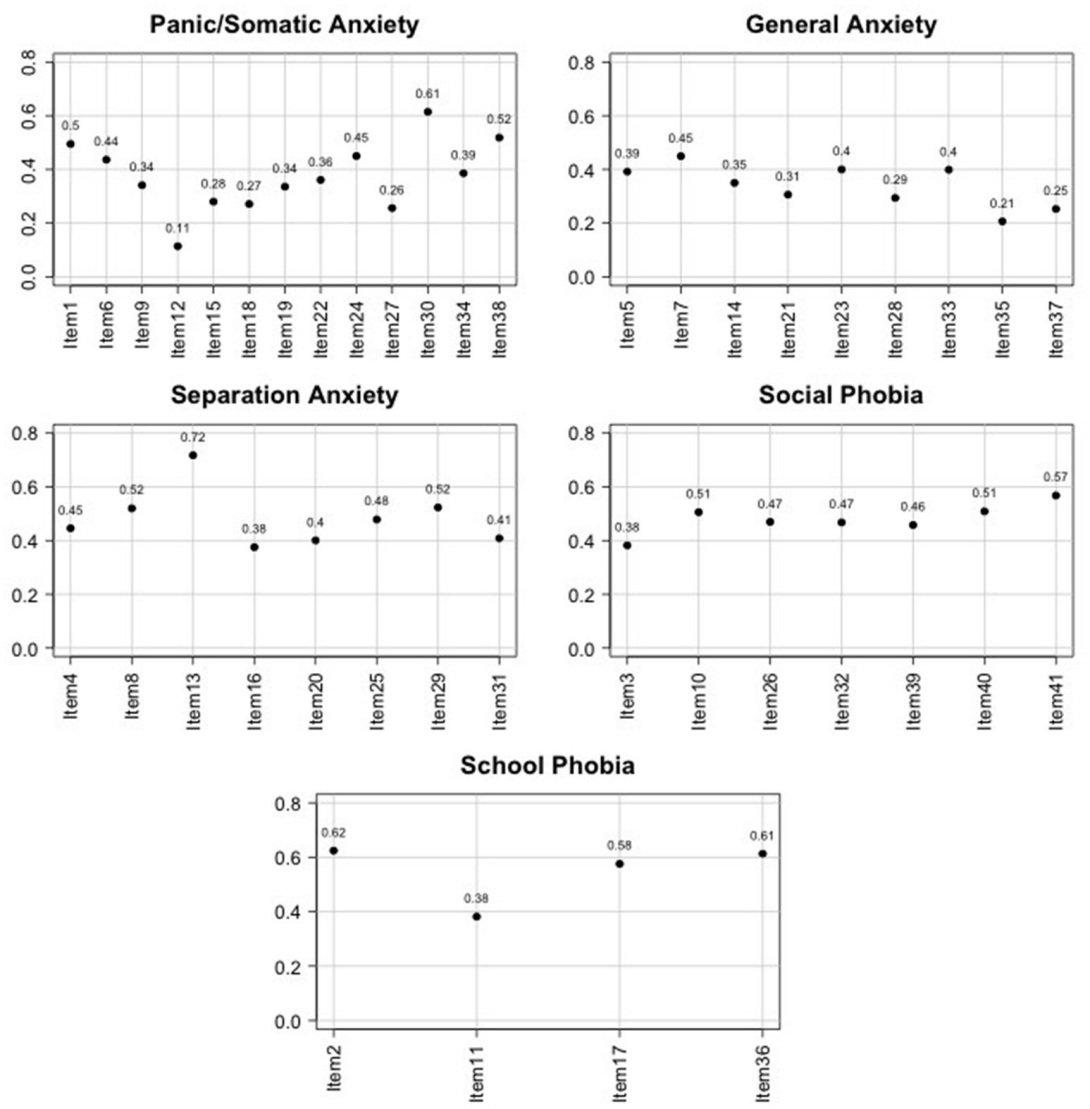
**Graphical representation of Cohen's κ for mother-child agreement within each item and for each SCARED subscale**.

A weighted kappa index with squared weights was calculated to evaluate mother-child agreement for each item belonging to a certain subscale (see Figure 1). κ values for the items of the PD scale range between 0.11 and 0.61 (from slight to substantial), values for items in the GAD scale range between 0.21 and 0.45 (from slight to moderate), for items included in the SAD scale agreement indices range between 0.38 and 0.72 (from fair to substantial), for items in the SOC scale κ-values range between 0.38 and 0.57 (from fair to moderate), and finally for the SCH scale they range between 0.38 and 0.62 (from fair to substantial). It seems that there is more agreement in the separation anxiety disorder scale.

### Comparison of factor structure analyses

We perform a factor analysis on polychoric correlation matrices of both parent and child version of SCARED questionnaire (Table 2).

**Table 2:** **SCARED items description both in English and Italian**.

	**Item English/Italian**
1	When I feel frightened, it is hard to breath/*Quando mi sento impaurito/a, faccio fatica a respirare*
2	I get headaches when I am at school/*Quando sono a scuola mi viene mal di testa*
3	I don't like to be with people I don't know well/*Non mi piace trovarmi con persone che non conosco bene*
4	I get scared if I sleep away from home/*Mi sento impaurito/a se devo dormire fuori casa*
5	I worry about other people liking me/*Mi preoccupo di piacere alle altre persone*
6	When I get frightened, I feel like passing out/*Quando vengo preso/a dalla paura mi sento come morire*
7	I am nervous/*Sono nervoso/a*
8	I follow my mother or father wherever they go/*Seguo mamma o papà ovunque vadano*
9	People tell me I look nervous/*La gente mi dice che sembro nervoso/a*
10	I feel nervous with people I don't know well/*Mi sento nervoso/a con le persone che non conosco bene*
11	I get stomachaches at school/*Quando sono a scuola mi viene mal di pancia*
12	When I get frightened, I feel like I am going crazy/*Quando vengo preso/a dalla paura mi sento come impazzire*
13	I worry about sleeping alone/*Ho paura di dormire da solo/a*
14	I worry about being as good as other kids/*Mi preoccupo di essere bravo/a come i miei coetanei*
15	When I get frightened, I feel like things are not real/*Quando vengo preso/a dalla paura mi sembra che le cose non siano vere*
16	I have nightmares about something bad happening to my parents/*Ho degli incubi che accada qualcosa di brutto ai miei genitori*
17	I worry about going school/*Mi preoccupa andare a scuola*
18	When I get frightened, my heart beats fast/*Quando vengo preso/a dalla paura il mio cuore batte forte*
19	I get shaky/*Mi viene da tremare*
20	I have nightmares about something bad happening to me/*Ho degli incubi che mi possa capitare qualcosa di brutto*
21	I worry about things working out for me/*Mi preoccupo che le cose non vadano per il verso giusto*
22	When I get frightened, I sweat a lot/*Quando vengo preso/a dalla paura sudo molto*
23	I am a worrior/*Sono uno/a che si preoccupa*
24	I get really frightened for no reason at all/*Mi spavento molto senza ragione*
25	I am afraid to be alone in the house/*Ho paura di rimanere solo/a in casa*
26	It is hard for me to talk with people don't know well/ *Faccio fatica a parlare con persone che non conosco bene*
27	When I get frightened, I feel like I am chocking/*Quando vengo preso/a dalla paura mi sento come strozzare*
28	People tell me that I worry too much/*Le persone mi dicono che mi preoccupo troppo*
29	I don't like to be away from my family/*Non mi piace stare lontano dalla mia famiglia*
30	I am afraid of having anxiety (panic) attacks/*Ho paura di avere attacchi d'ansia (o di panico)*
31	I worry that something bad might happen to my parents/*Ho paura che possa accadere qualcosa di brutto ai miei genitori*
32	I feel shy with people that I don't know well/*Sono timido/a con le persone che non conosco bene*
33	I worry about what is going to happen in the future/*Mi preoccupo di quello che succederà in futuro*
34	When I get frightened, I feel like I trowing up/*Quando vengo preso/a dalla paura mi sento come se dovessi vomitare*
35	I worry about how well I do things/*Mi preoccupo di quanto riesco a fare bene le cose*
36	I am scared to go to school/*Ho paura di andare a scuola*
37	I worry about things that have already happened/*Mi preoccupo di cose che sono già successe*
38	When I get frightened, I feel dizzy/*Quando vengo preso/a dalla paura mi vengono le vertigini*
39	I feel nervous when I am with other children or adults and I have to do something while they watch me (for example: read aloud, speak, play a game, play a sport)/*Mi sento nervoso/a quando sono con altri coetanei o adulti e devo fare qualcosa mentre loro mi guardano (ad esempio, leggere a voce alta, parlare, giocare, fare sport)*.
40	I feel nervous when I am going to parties, dances, or any place where there will be people that I don't know well/*Mi sento nervoso/a all'idea di andare alle feste, a ballare o in qualsiasi posto dove ci sono persone che non conosco bene*
41	I am shy/*Sono timido/a*.

In the current literature, only Wren et al. (2007) evaluated differences between parent and child questionnaire structures, using separate principal component analyses and reporting a four factor solution for both versions largely replicating the original factor structure found in Birmaher et al. (1997, 1999), where the school subscale did not emerge.

**Figure 2:**
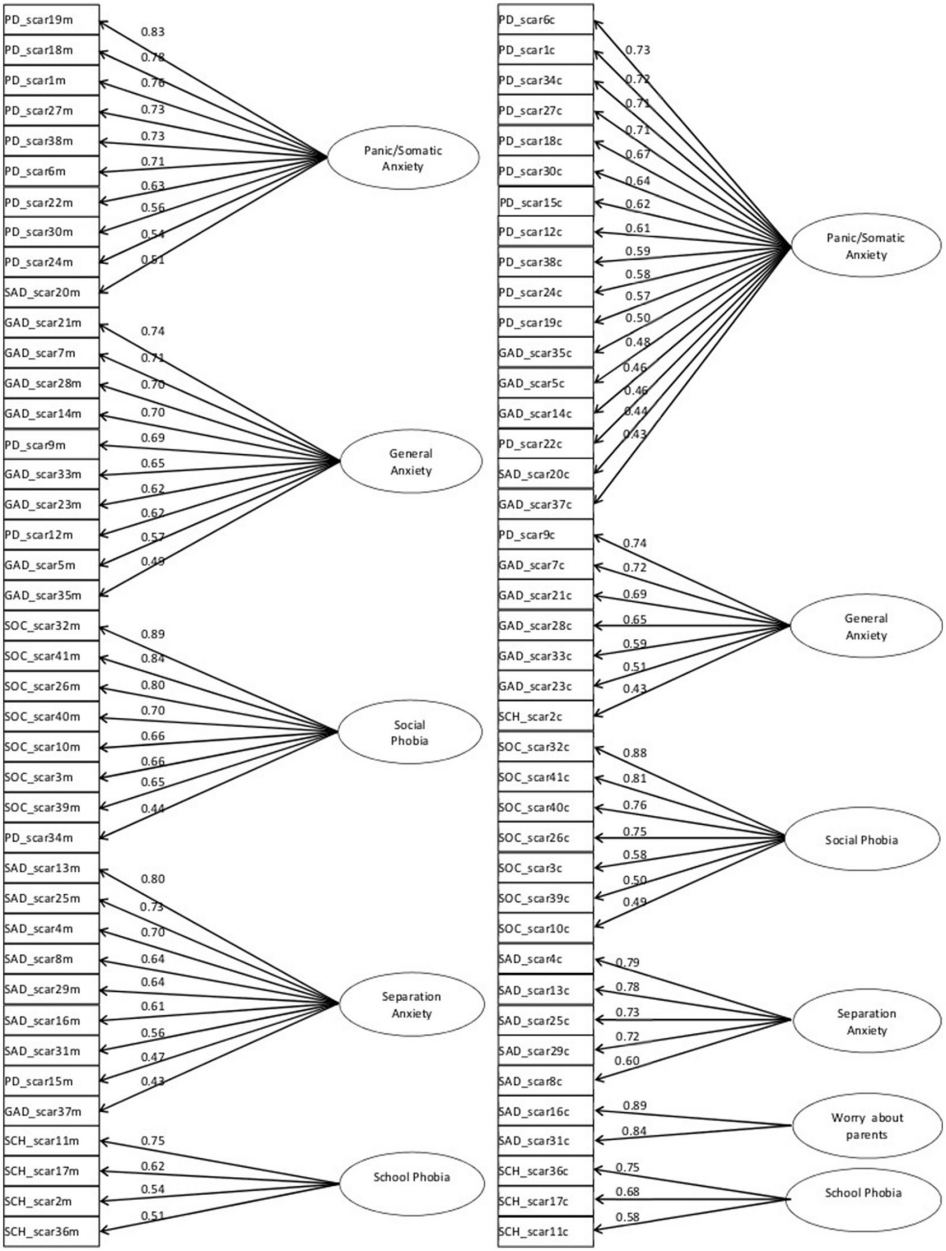
**Diagrams representing factor structure for mother (left)** and child **(right)** SCARED questionnaires obtained through exploratory factor analysis for categorical data.

In our data, parallel analysis on SCARED parent version, completed by the mothers (*n* = 126, complete observations), suggested a 5 factor solution (Figure 2). Velicer's MAP criterion and empirical Bayesian Information Criterion (eBIC) confirmed this solution, while VSS criteria (complexity 1, complexity 2) were more conservative, suggesting 1 or 2 factors. However, caution is adviced when interpreting VSS criterion since it is not always optimal and simulation results have shown that this criterion is not appropriate when data shows a complex factor structure (Revelle, 2014).

The Root Mean Square of the Residuals (RMSR) for the 5 factor solution was 0.07. Five factors explained 62% of the variance.

For the sake of interpretability, factor loadings were orthogonally rotated using the Varimax method. Oblique rotation was not appropriate in our case since within-factors correlations were very low.

Based on the items that load on each factor, the first factor was named “Panic/Somatic Anxiety” (it includes 9 out of the 13 items that make up the PD scale), the second one was named “Social Phobia” (all the items that make up the SOC scale originally proposed in Birmaher et al., 1997), the third “Separation Anxiety” (7 out of the 8 items that construct the SAD scale), the fourth “General Anxiety” (8 out of the 9 items that construct the GAD scale) and the last “School Phobia” (all the items that make up the school phobia scale).

Parallel analysis on SCARED child version, completed by *n* = 147 children in total (considering complete records), suggested a 4 factor solution. Velicer's MAP criterion suggested a 3 factor solution, eBIC a 6 factor solution, while VSS criteria (complexity 1, complexity 2) suggested 1 and 4 factors, respectively.

We explored a range of solutions going from 4 to 6 factors. Actually we choose a 6 factor solution as best, explaining 60% of the total variance (Figure 2). The RMSR was 0.08. Again, orthogonal (Varimax) rotation was used.

Based on the items that load on each factor, the first factor was named “Panic/Somatic Anxiety” (it includes 12 out of the 13 items that make up the PD scale in the solution proposed by Birmaher et al., 1997), the second “Separation Anxiety” (5 out of the 8 items included in the original SAD scale proposed in Birmaher et al., 1997), the third was named “Social Phobia” (all the items that make up the SOC scale originally proposed), the fourth “General Anxiety” (5 out of the 9 items that construct the GAD scale), the fifth including the 2 items of “Separation Anxiety” subscale associated with a possible danger for parents was named “Worry about Parents” and the last was called “School Phobia” (3 out of the 4 items originally that make up the school phobia scale).

## Discussion and conclusion

Although structured interviews still represent the gold standard to determine the presence and degree of severity of a clinical disorder, the use of reliable and valid self-reported questionnaires may support clinicians in the diagnosis and may be used as screening tools for children (Alfano et al., 2002; Viana et al., 2008).

The present study represents the first contribution that evaluates cross-informant agreement of the Italian child and parent versions of SCARED. Moreover, statistical approaches specifically suited to ordinal data have been applied.

Our results confirmed hypotheses that children report significant information about anxiety symptoms and thus contribute with a different perspective to the assessment of the disorder.

Our analysis shows a moderate to strong mother-child agreement. When accounting for age, we found that the level of cross-informant agreement for the total SCARED score increased with age. This can be interpreted in the following way: as children grow up, they become more able to communicate and explain their symptomatology to parents who in turn become more aware of the child's anxiety. Our results suggested that, independently on age, the subscale with the higher rate of agreement is SAD. From a clinical perspective, this reflects the fact that SAD symptoms emerge clearly in the family since the child would often ask for help and assurances. In addition, SAD symptoms compromise family life and increase parental stress limiting the activities of siblings and parents (Fischer et al., 1999). In fact, SAD's essential feature is an excessive anxiety concerning separation of the child from its home or parents (American Psychiatric Association, 2013). Differently from other anxiety disorders, the fear of separation causes great distress to the child and may interfere with both the child's and the family's normal activities (Fischer et al., 1999). A child suffering from SAD may often show avoidance and oppositional behavioral patterns in an attempt to prevent separation, or may refuse to participate in activities requiring separation from the attachment figure (Fischer et al., 1999).

Moreover, current studies (e.g., Scaini et al., 2012) suggest that separation anxiety develops from multiple and interrelated factors that include genetic vulnerabilities and environmental factors, such as negative family experiences (Chorpita, 2001; Kearney, 2008).

Our results appear consistent with recent research (Wren et al., 2004; Su et al., 2008; Weitkamp et al., 2010; Jastrowski Mano et al., 2012) that reports agreement values higher than the ones previously reported by Birmaher et al. (1997, 1999).

Despite evidence of good cross-informant agreement in the present study, strong differences in number of reported symptoms and their severity emerged from the analyses.

When accounting for the age effect, we observe that children reported on average more symptoms of Separation Anxiety at 8–10 years and of General Anxiety at 16–18 years. Moreover, mean scores in General Anxiety scale increase with age.

The bulk of excessive symptoms reported by children was for Somatic/Panic Anxiety and General Anxiety and came from girls. These results are consistent with other reports investigating parent-child agreement (Wren et al., 2004) and with epidemiological studies that reported higher rates of anxiety symptoms for girls (Kessler et al., 2005; Ogliari et al., 2006; Beesdo et al., 2009). However, we found similar mother-child agreement when stratifying by gender. This suggests that mother-son and mother-daughter accord is similar.

Following the approach of Birmaher et al. (1997) we separately analyzed parent- and self-report versions of the SCARED.

Differently from all the recent studies that have evaluated psychometric properties of the SCARED, an exploratory factor analysis for ordinal data was carried out on polychoric correlation matrices calculated on SCARED items. This approach has been recommended in the literature to properly analyze Likert type ordinal items (Muthén and Kaplan, 1985; Gilley and Uhlig, 1993; Jöreskog and Moustaki, 2001) with factor analysis procedures. Simulation studies showed that the solutions obtained using polychoric correlations provide a more accurate reproduction of the measurement model used to generate the data (Holgado-Tello et al., 2010).

Two different factorial solutions emerged for parent and child SCARED versions. Analyses on parent version revealed a 5 factor structure that approximately reproduces the original structure proposed by Birmaher et al. (1997, 1999) and by other authors (e.g., Jastrowski Mano et al., 2012) for parent version. Based on our findings, a 6 factor solution was chosen as best in the child version, with the first 5 factors reproducing “Birmaher” factors, whereas the 6th include the two items of Separation Anxiety subscale associated with a possible danger for parents (16. I have nightmares about something bad happening to my parents/31. I worry that something bad might happen to my parents). This result suggests that, in children, anxiety concerning of “being alone” or “a possible danger for children” emerged separately from anxiety about a possible danger for parents.

A limitation of the present study concern sample size. Indeed, for factor analysis, we considered SCARED questionnaire completed by 126 mothers and 147 children. However, when analyzing age effects in the evaluation of mother-child agreement, sample size within each age category is unbalanced and some categories are not well-represented. In particular, descriptive statistics for the SCARED scales are actually based on the information provided by 49 children in the age-class category 8–10 yrs, 53 children in the age-class category 11–13 yrs, 34 children in the age-class category 14–15 yrs and 35 in the last category 16–18.

In future work, the current analysis could be enhanced by addressing the following issues.

Data on parent reports were gathered from mother alone: analyses integrating father ratings may yield more informative results thus providing greater generalizability.

Future research should focus on longitudinal study designs, thus allowing test-retest reliability evaluations.

Hence, to conclude, our findings could help clinicians to determine which information, and from which rater, must be accounted for in evaluating treatment decisions. Moreover, due to the emerging differences in reported symptoms and cross-informant agreement depending on respondent's age, clinicians should consider potential age effect in interpreting SCARED results during assessment process.

## Author contributions

SS and CB conceived of the study, participated in its design and coordination and drafted the manuscript; CB performed the statistical analysis; AO, CD, and LB participated in the design, coordination of the study and helped to draft the manuscript; LD contributed to literature collection and helped with data analysis. All authors read and approved the final manuscript.

## Funding

This research has been funded by the FIRB Project RBFR12VHR7 entitled “Interpreting emotions: a computational tool integrating facial expressions and biosignals based on shape analysis and Bayesian networks.”

### Conflict of interest statement

The authors declare that the research was conducted in the absence of any commercial or financial relationships that could be construed as a potential conflict of interest.
